# Qualitative interviews to improve patient-reported outcome measures in late-onset Pompe disease: the patient perspective

**DOI:** 10.1186/s13023-021-02067-x

**Published:** 2021-10-12

**Authors:** Alaa Hamed, Kristina An Haack, Chad Gwaltney, Eileen Baranowski, Andrew Stewart, Robert Krupnick, Margaret Tyler, Susan Sparks, Jean Paty

**Affiliations:** 1 Sanofi Genzyme, 50 Binney Street, Cambridge, MA 02142 USA; 2 Sanofi Genzyme, Shanghai, China; 3 Gwaltney Consulting, Westerly, RI USA; 4 IQVIA INC, New York City, NY USA

**Keywords:** Late-onset Pompe disease, PROs, Conceptual model, Symptoms, Patient experience, Functional impacts, Lysosomal storage disorders

## Abstract

**Background:**

Late-onset Pompe Disease (LOPD) is a rare, heterogeneous disease manifested by a range of symptoms varying in severity. Research establishing the frequency of these symptoms and their impact on patients’ daily lives is limited. The objective of this study was to develop a conceptual model that captures the most relevant symptoms and functional limitations experienced by patients with LOPD, to inform the development of new patient-reported outcome (PRO) tools.

**Methods:**

A preliminary conceptual model was constructed following a literature review and revised through interviews with expert clinicians to identify important and relevant concepts regarding symptoms and impacts of LOPD. This preliminary model informed the development of a qualitative patient interview guide, which was used to gather the patient perspective on symptoms and impacts relating to LOPD or its treatment (including symptom/impact frequency and levels of disturbance). Patient interviews aided further refinement of the conceptual model. The findings from the patient interviews were triangulated with the literature review and clinician interviews to identify the most relevant and significant effects of LOPD from the patient perspective.

**Results:**

Muscle weakness, fatigue, pain, and breathing difficulties (especially while lying down) were the most common and highly disturbing symptoms experienced by patients. Limitations associated with mobility (e.g., difficulty rising from a sitting position, getting up after bending) and activities of daily living, (e.g., reduced ability to participate in social/family activities or work/study) were the most frequently reported impacts with the highest levels of disturbance on the patient’s daily life. These identified symptoms and impacts were included in the new conceptual model of disease.

**Conclusions:**

This qualitative patient interview study, also informed by a literature review and clinician interviews, identified the most frequent and relevant symptoms and the functional impact of LOPD on patients. The study interviews also captured the patient-preferred language to describe symptoms and impacts of LOPD. The results from this study can be used to develop future PRO instruments that are tailored to the specific symptoms and impacts experienced by patients with LOPD.

**Supplementary Information:**

The online version contains supplementary material available at 10.1186/s13023-021-02067-x.

## Background

Pompe disease (PD) is a rare, heterogeneous neuromuscular disorder in which patients can present with a range of symptoms with varying degrees of severity. PD is also known as glycogen-storage disease type II (GSD2) or acid maltase deficiency [1]. PD results from pathogenic *GAA* gene variants which cause a decrease or absence of acid α-glucosidase (GAA) enzyme activity, progressive lysosomal glycogen accumulation in certain tissues, and skeletal muscle dysfunction [1, 2]. Although considered a rare disease, with an estimated frequency of one in 40,000, high prevalence rates of PD are found in some populations [3–5].

Clinically, PD can present with a range of phenotypes, ranging from the rapid progressive infantile-onset form of the disease (IOPD) to the slower, progressive late-onset (LOPD) form [6, 7]. Literature reports a range of diverse symptoms of LOPD which can manifest at any age, with better prognosis reported for patients who present with later symptoms onset [7]. Patients with LOPD experience a steady degeneration of respiratory and skeletal muscle function, as well as involvement of the gastrointestinal, vascular, and cardiac systems [1, 7–10]. A review of 225 published cases of PD revealed that muscle weakness was commonly one of the initial symptoms of disease [7]. Adult patients with LOPD typically develop respiratory and skeletal muscle dysfunction leading to a need for assisted ventilation and loss of ambulation, while infantile hypertrophic cardiomyopathy in IOPD can lead to cardiorespiratory failure [1, 7]. The main reason for morbidity and mortality in adults with LOPD is a progressive respiratory muscle weakness [11]. Enzyme replacement therapy (ERT) with alglucosidase alfa, which has been available since 2006, has been shown to improve the physical health of patients with LOPD and their ability to perform daily activities [12–14]. However, despite treatment, patients still experience symptoms and limitations [15]. Although it is recognized that patients with LOPD have a lower quality of life due to their reduced physical health status and limitations in their ability to work [12, 16], little is definitively known about the frequency and impact of the symptoms of LOPD on patients’ daily lives.

The quantification of the effects of PD regarding activities of daily living (ADL) and social participation can be accurately reported using the 18-item Rasch Built Pompe Specific Activity (R-PAct) scale [17]. A 5-year prospective study of 102 patients with PD showed how the R-PAct score generally improved for participants during ERT and was higher after 5 years compared with baseline values on initiation of ERT [18]. However, the R-PAct is limited as it does not measure the respiratory symptoms of PD. Another scale, the Rotterdam Handicap Scale (RHS) designed to assess handicap or independence in patients with neuromuscular disorders, can also be used to assess the impact of LOPD on ADL; however, it is not disease-specific [19]. In a study of 257 adult patients with LOPD, results showed that the mean RHS score differed significantly between patients with and without respiratory support, indicating that respiratory symptoms and impacts (symptom burden) are important to address as disease-specific items [20]. A recent conceptual model for adult PD using the Wilson-Cleary health outcomes model as a framework [21], was developed to address quality of life in untreated patients with PD [22]. Patient-reported outcome (PRO) data were acquired from multiple patient observations during standardized medical follow-up examinations. In this patient population, regression analyses revealed that patients’ functional status was affected by fatigue, muscle strength and respiratory function [22].

Implementation of a patient-centered approach to both clinical research and care settings in recent years has increased the recognition of PRO measures as informative and reliable tools for health-related quality of life assessment. However, data on the use of PRO measures to evaluate the impact of therapy on LOPD symptoms and experiences are limited, and additional research may identify patient experiences that can directly support the development of PRO items for use in clinical trials. Although studies have been performed to look at activities and symptoms in patients with LOPD with quantitative measures, there is a need for qualitative data acquired through focus groups and interviews to further develop LOPD-specific PRO measures, to ensure that the PRO items generate data that provide an unbiased and comprehensive description of a patient’s experience [23]. A review of PRO or observer-reported outcome measures addressing health-related quality of life in inherited metabolic diseases reported that of 32 measures identified only 2% were disease-specific. These results highlight the need for developing novel disease-specific outcome measures that are sensitive to the patient perspective during clinical research [24].

The aim of this study was to interview patients with LOPD to obtain an in-depth understanding of their daily lives, including the symptoms and functional limitations that they experience. This study also aimed to address the specific language used by patients when describing how the disease affects their daily living. LOPD symptoms and functional impacts were prioritized to determine those most relevant to people living with LOPD, to facilitate the development of PRO instruments for this underserved population.

## Methods

This was a qualitative study in which a preliminary conceptual model (based on literature reviews and expert clinician feedback) was developed to identify important and relevant concepts regarding symptoms and impacts of LOPD. This model was used to inform a concept elicitation interview guide for patients to gather their perspective on symptoms and impacts relating to LOPD or its treatment, and to further revise the conceptual model.

### Development of the preliminary conceptual model

A preliminary conceptual model was developed to identify the most relevant LOPD signs, symptoms, and functional limitations, informed by published literature and instrument reviews. Subsequently, three expert clinicians, who each saw up to 70 patients with PD per year, were recruited to revise the preliminary conceptual model; the clinicians practiced at university medical centers across neurology, pediatrics, genetics/metabolism, and physical medicine and rehabilitation departments.

Refinement of the preliminary conceptual model was made through identification of concepts (or themes) to be added, removed, altered, prioritized, or deprioritized. The expert clinicians also provided additional input regarding the burden associated with symptoms of LOPD. The preliminary model, and understanding of best practices for how to conduct PRO interviews, informed the development of a revised interview discussion guide to be used in subsequent patient interviews.

### Patient screening and eligibility criteria

Patients were identified, consented, and screened in collaboration with the Acid Maltase Deficiency Association (AMDA), an advocacy group devoted to patients with PD. The advocacy organization was aware that the research study was funded by Sanofi Genzyme and did not receive payment or any other recompense for its cooperation. The AMDA disseminated an introductory letter to all its members via email, which included the purpose of the research study, a link to a programmed consent document, and a screener survey instrument. AMDA maintained patient privacy and no contact information was shared with Sanofi Genzyme. Consenting participants received an online screening form that included questions related to patient demographics, symptoms, diagnosis, and treatment to confirm that they met eligibility requirements.

Eligible participants were patients with LOPD who confirmed they were receiving ERT for their condition. Eligibility criteria also stipulated a requirement to speak clearly in English, for patients to reside in any State within the U.S.; patients could be of either gender and ≥ 18 years of age, or ≥ 19 years of age in the States of Alabama or Nebraska. Patients were excluded from this study if they were currently participating in another clinical trial, were physically unable to participate in a 60-min telephone interview, or if they used a ventilator full-time. Patients were also excluded if they, or any family member, were currently affiliated with the U.S. Food and Drug Administration (FDA) or any Government agency that approves medications, an advertising agency, a marketing research company, or a pharmaceutical or biotechnology company. Participants were compensated for their time and effort in taking part in the interview with a gift card.

### Patient interviews

Thirteen interviews with patients with LOPD were conducted in three rounds, with 4–5 patients in each round sequenced from their date of participation in the study. Patients were interviewed by experienced (> 3 years) trained moderators (1 male and 1 female) via telephone using a semi-structured interview guide to explore patients’ perspectives on the key symptoms and impacts of LOPD and further inform the conceptual model. Anonymised transcripts were produced from all recorded interviews. During the interview, patients were asked to rate the level of disturbance related to each symptom they experienced on a 0–10 numeric rating scale (with 0 = “not disturbing at all” to 10 = “extremely disturbing”). Each symptom or impact detail (whether elicitation was aided or unaided), the patient’s description of their symptoms in their own words (copied directly from the transcripts), the symptom or impact severity, the disturbance scores, and the frequency of occurrence, were recorded.

Table 1 provides details of questions included in the patient interview guide. Questions were open-ended to avoid bias and were not read verbatim to permit free flowing discussion. The guide included prompts to direct the interviewer in probing patient experiences in greater depth. Patient responses were captured by the interviewer on an anonymized copy of the guide as well as on anonymized worksheets for reported symptoms and symptom impacts. Sessions were audio recorded with the permission of the patient.

**Table 1:** The topics discussed in concept elicitation interviews

Topic in concept elicitation interview	Objective
LOPD symptoms: Asked about timing and triggers of symptoms Asked about how symptoms have evolved over time Asked about symptom characteristics (spontaneous report, then aided) and whether the patient felt that the symptom(s) was related to treatment	To assess frequency of symptoms reported, level of disturbance for each symptom, words and phrases patients use to report symptoms, and possible differences in patient experience over time
LOPD impacts: Asked about the kinds of impacts or effects of the symptoms on the patient’s life Asked about the degree of disturbance of these impacts on the patient’s life	To assess frequency of impacts reported, level of disturbance for each impact, words and phrases patients use to report impacts, and possible differences in patient experience over time

*LOPD* late-onset Pompe disease

The first two rounds of interviews were performed to gain a general understanding of the symptoms and symptom impacts experienced by patients with LOPD (Groups 1 and 2). The third round of interviews with patients (Group 3) was to confirm reasonable concept saturation of symptoms and impacts which were important to patients, and to ensure that no relevant concepts had been missed. In an attempt to aid patients in their responses and at the request of the sponsor, or due to variations offered by patients in the first two groups of interviews, minor alternations in wording of the interview probes were applied to the third group of interviews (i.e., ‘Trouble breathing while lying down’ was changed to ‘Difficulty breathing while lying down’). In each case, careful consideration was applied by the team of analysts before grouping with previous versions. For a complete list of text changes see Additional file 1.

### Data synthesis and analysis

To organize the patient expressions, a coding framework was developed for use with ATLAS.ti software (ATLAS.ti Scientific Software Development GmbH), so that patient statements reflecting similar concepts were grouped together, for example, patient statements of “I have trouble breathing when I exert myself” and “sometimes I can’t catch my breath” would both be categorized as ‘breathing difficulties.’ The coding frame formed the basis for grouping concepts using an adaptation of grounded theory to incorporate prior clinical knowledge based on the scientific literature and expert clinical opinion. This permitted trained qualitative analysts (coders) to move back and forth between hypothetic-deductive and inductive approaches. Coders identified text that included concept expressions and tagged this text with a code from the coding frame. The coding frame was updated continuously throughout the coding process as concepts developed and new concepts arose. New codes were retroactively applied to previously coded transcripts at the time point at which the new codes occurred.

Descriptive analysis was performed to determine the relative frequency of symptoms and impacts, and ratings of average disturbance of symptoms and impacts, as well as any additional characteristics that further described the patient experience. Anonymized transcripts of the patient interviews were also used to form a listing of the specific words and phrases that patients used during the interviews, using the categories addressed in the interview guide, e.g., mobility problems and muscle weakness. To cross-verify and, therefore, validate the study results, data obtained from patient interviews were triangulated with those gained from both the literature review and the expert interviews.

There is no formal statistical methodology to prospectively estimate sample size targets for qualitative research similar to the approaches available for quantitative research. The accepted marker of adequacy is when a sufficient number of the target population has been interviewed to reach a point where no new information is forthcoming, known as “saturation of concept”. In a rare condition, such as PD, saturation of concept may be defined more flexibly, with the aim of identifying the full range of key concepts (categories and sub-categories deemed as particularly important to patients, i.e., what is being measured) [25–27]. Therefore, to determine saturation of LOPD symptom-related concepts and corresponding impacts, the total sample size was divided into three different sub-groups (Groups 1–3) determined by date of recruitment interview. The concepts derived from each group were compared with those in the previous group to identify the emergence of new concepts. Assessments were made of symptom and impact concept saturation in line with International Society for Pharmacoeconomics and Outcomes Research (ISPOR) guidelines [27].

## Results

A total of 26 individuals expressed interest in participating in the study and were sent an online screening form. Of those, eight who had originally expressed interest did not take further steps to participate once they received a screening form, and five were ineligible for the study as they did not meet one or more of the inclusion criteria. There were no dropouts during the study.

The first two rounds of patient interviews (Groups 1 and 2; both n = 5) were conducted in March and April 2015, and the third round (Group 3; n = 3) in August 2015. Patient demographics and clinical information are summarized in Table 2. Mean (SD) age was 56 (13) years (range 31–71 years) and there were marginally more males (54%) than females (46%). On average, patients were diagnosed with LOPD 12 years prior to the interview (median [range] 8 [1–34] years).

**Table 2:** Demographic characteristics and clinical information

Demographic characteristics and clinical information	Total (N = 13)
Age (years)	
Mean (SD)	56 (13)
Median (range)	60 (31 − 71)
Gender, n (%)	
Male	7 (54)
Female	6 (46)
Highest level of education completed, n (%)	
Some college or associate degree	12 (92)
Graduate school degree	1 (8)
Age at first symptoms (years)	
Mean (SD)	21 (12)
Median (range)	21 (3–40)
Age at first seeing a doctor for symptoms (years)	
Mean (SD)	38 (13)
Median (range)	40 (14–59)
Years between first symptoms and seeing a doctor	
Mean (SD)	17 (12)
Median (range)	13 (0–29)
Age at diagnosis (years)	
Mean (SD)	45 (14)
Median (range)	49 (22–63)
Years between diagnosis and seeing a doctor	
Mean (SD)	7 (5)
Median (range)	7 (1–15)
Years since diagnosis	
Mean (SD)	12 (10)
Median (range)	8 (1–34)

*SD* standard deviation

**Fig. 1:**
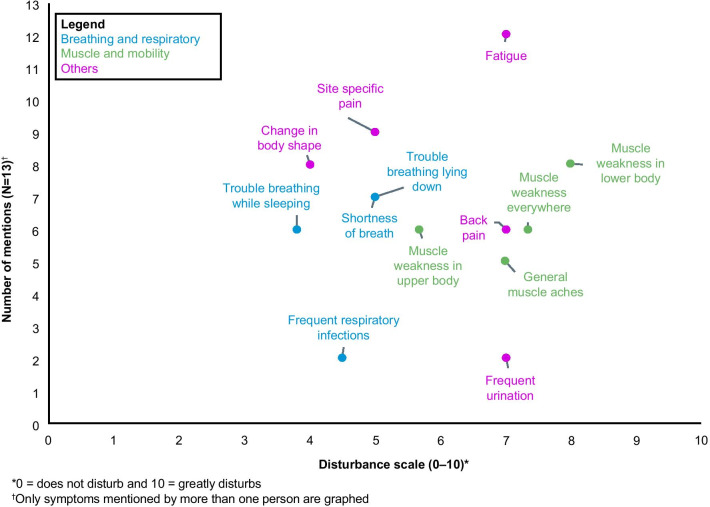
Summary of patient findings: symptoms

Patient-reported symptoms and their frequency are reported in Table 3 (Additional file 2 provides details of patient interview quotes). The symptoms most frequently reported were ‘fatigue’ (92%): “Probably the worst one [symptom] would be fatigue. That’s always there…Feeling tired all the time…”; and ‘site-specific muscle pain’ (69%): “The stabbing and shooting pains are usually in my legs and feet, sometimes in my arms, and there’s a lot of achy pain in my back. Mostly my lower back, but sometimes it’s all over…”*.* A majority of these patients also described ‘muscle weakness in lower body (hips and/or legs)’ (62%), stating “I say it’s a muscle weakening disease…Muscle weakness is always there. Like it’s impossible to bend over and pick up something from the floor and stand up again. Even if it’s a tissue or something very light” and ‘change in body shape’ “A Pompe pouch, a lot of people call it that. Where we get a little bit of a bloated belly” (62%). In addition, ‘back pain’ was reported by 54% of patients: “…Mostly my lower back, but sometimes it’s all over. And when I fall, my tailbone usually hurts for a good three, four, five days afterwards…”. Breathing and respiratory symptoms were also reported, with 54% of patients describing ‘shortness of breath’: “…You just feel like you’re not getting enough air, I guess” and ‘trouble breathing while lying down’: “When I go to sleep or when I lay down it feels like [something is] on my chest you know and it’s just, it’s hard to breathe”*.* Patient-reported symptom disturbance ratings (mean [SD]) are reported in Table 3; ‘muscle weakness in lower body (hips and/or legs)’ (8.0 [2.1]), ‘muscle weakness everywhere’ (7.3 [1.8]), and ‘fatigue’ (7.3 [1.5]) were the symptoms that patients with LOPD considered as the ones that disturbed or affected daily living the most. The patient-based evidence for symptom-related experiences considered as relevant and important to patients diagnosed with LOPD are summarized in Fig. 1. Overall, patients experienced a small, concentrated group of symptoms that had similar levels of disturbance and frequency of mentions.

**Table 3:** Frequency of LOPD symptoms and associated symptom disturbance rating

Symptom concepts descriptors for LOPD	Total mentions (N = 13)	Symptom disturbance, mean (SD) (0 = not disturbing at all; 10 = extremely disturbing)
Muscle and mobility		
Site-specific pain (not back) *Pain	9 (69.2%)	5.3 (2.5)
Muscle weakness in lower body (hips and/or legs)	8 (61.5%)	8.0 (2.1)
Change in body shape	8 (61.5%)	4.4 (3.1)
Back pain	7 (53.8%)	6.3 (2.4)
Muscle weakness in upper body (core and/or arms)	6 (46.2%)	6.4 (2.1)
Muscle weakness everywhere	6 (46.2%)	7.3 (1.8)
Muscle aches	5 (38.5%)	6.6 (3.1)
Stabbing, shooting, sharp pain	1 (7.7%)	1.0 (0.0)
Moderate to severe scoliosis	1 (7.7%)	5.0 (0.0)
Breathing and respiratory		
Shortness of breath *Breathing difficulties	7 (53.8%)	4.5 (2.5)
Trouble breathing while lying down *Breathing difficulties while lying down	7 (53.8%)	5.4 (3.7)
Trouble breathing while sleeping	6 (46.2%)	3.8 (2.5)
Frequent respiratory infections	2 (15.4%)	4.5 (0.5)
Trachea secretion and drainage	1 (7.7%)	5.0 (0.0)
Other		
Fatigue *Tiredness/fatigue/need to rest	12 (92.3%)	7.3 (1.5)
Frequent urination/OAB	2 (15.4%)	6.5 (0.5)
Acid reflux	1 (7.7%)	2.0 (0.0)
Loose bowel	1 (7.7%)	8.0 (0.0)
Some fiber neuropathy	1 (7.7%)	7.0 (0.0)
Stiffness in back	1 (7.7%)	9.0 (0.0)
Headaches *Headaches *Morning headaches	1 (7.7%)	4.0 (0.0
Hives related to infusion	1 (7.7%)	-

*LOPD* late-onset Pompe disease, *OAB* overactive bladder, *SD* standard deviation

*Denotes wording applied to third round of interviews only (Additional file 1)

Patient-reported impacts, frequencies, and impact disturbance ratings are reported in Table 4. Overall, five symptom-related experiences were recorded as having the most impact on this cohort of patients with LOPD: ‘reduced ability to perform household tasks’ (92%), ‘reduced ability to participate in leisure activities I used to be able to’ (85%), ‘reduced ability to participate in social activities’ (85%), ‘unwanted weight gain/difficulty losing weight’ (77%), and ‘difficulty rising from an armchair’ (77%). Furthermore, a cluster of specific disease-related effects regarding decreased mobility and inability to rise from a sitting position were described by patients. “I can’t bend very far or I’ll be down on the ground… If I have to pick anything up, it’s a struggle…”*.*

**Table 4:** Frequency of impacts in LOPD and associated disturbance rating

Impact concepts descriptors for LOPD	Total mentions (N = 13)	Impact disturbance, mean (SD) (0 = not disturbing at all; 10 = extremely disturbing)
Muscle and mobility		
Difficulty rising from an armchair *Difficulty rising from a sitting position	10 (76.9%)	6.8 (2.6)
Difficulty with stairs (primarily going up) *Difficulty climbing stairs	9 (69.2%)	7.0 (3.1)
Change in the way you walk (“Pompe waddle”)	8 (61.5%)	5.5 (3.4)
Loss of balance	8 (61.5%)	5.9 (2.6)
Difficulty getting upright after bending	8 (61.5%)	5.8 (3.0)
Difficulty rising from a squatting position	8 (61.5%)	6.5 (4.1)
Difficulty playing sports	7 (53.8%)	7.3 (2.7)
Cannot walk without assistance *Can walk with assistance (from another person or a device, like a cane or walker)	6 (46.2%)	7.8 (1.7)
Reduced ability to move independently	5 (38.5%)	6.4 (2.9)
Cannot walk at all	4 (30.8%)	8.5 (1.7)
Can walk but slower and shorter distances than before	3 (23.1%)	6.7 (3.4)
Falling	2 (15.4%)	9.5 (0.5)
Difficulty getting up from a fall	2 (15.4%)	10.0 (0.0)
Cannot lift arms all the way up	1 (7.7%)	7.0 (0.0)
Difficulty walking in the dark	1 (7.7%)	8.0 (0.0)
Need to use standing frame	1 (7.7%)	–
Eating		
Unwanted weight gain/difficult losing weight *Unwanted weight gain *Difficulty losing weight	10 (76.9%)	5.8 (2.4)
Difficulty chewing, swallowing, etc	9 (69.2%)	3.7 (2.1)^†^
Cannot stay vegetarian	1 (7.7%)	4.0 (0.0)
Activities of daily living		
Reduced ability to perform household tasks	12 (92.3%)	5.4 (2.6)
Reduced ability to participate in leisure activities I used to be able to	11 (84.6%)	6.6 (2.3)
Reduced ability to care for oneself (showering, dressing, etc.)	5 (38.5%)	8.3 (1.2)
Cannot participate with family in their leisure activities	1 (7.7%)	10.0 (0.0)
Reduced/change in ability to write music	1 (7.7%)	7.0 (0.0)
Use “urine director” to go to the bathroom	1 (7.7%)	2.0 (0.0)
Need to be very regulated and methodical about daily activities/more planning required	1 (7.7%)	2.0 (0.0)
Reduced ability to participate in social activities *Reduced ability to participate in social/family activities	11 (84.6%)	5.4 (3.4)
Change in body image	7 (53.8%)	3.9 (1.9)
Depression	7 (53.8%)	3.6 (1.5)
Anxiety	6 (46.2%)	7.0 (2.2)
Feels like a burden to family/others	6 (46.2%)	4.5 (3.5)
Difficulty coping	2 (15.4%)	5.5 (2.5)
Reduced ability to effectively communicate	1 (7.7%)	4.0 (0.0)
Mom felt guilty because of genetic pre-disposition	1 (7.7%)	2.0 (0.0)
Change in what she can do as a wife and mother to help take care of her family	1 (7.7%)	2.0 (0.0)
Getting used to accepting the disease	1 (7.7%)	8.0 (0.0)
Worry about my future	1 (7.7%)	3.0 (0.0)
Other		
Financial difficulties	8 (61.5%)	6.8 (2.9)
Reduced ability to work/study	9 (69.2%)	7.0 (3.4)
Difficulty traveling (by car, bus, plane)	9 (69.2%)	5.9 (3.2)
Sexual issues (e.g., need to use different positions)	7 (53.8%)	5.7 (3.7)
Sleep problems (e.g., need to get up frequently)	6 (46.2%)	4.2 (2.5)
Difficulty being away from home	1 (7.7%)	8.0 (0.0)
Question whether able to take care of young children	1 (7.7%)	9.0 (0.0)
Uncertainty whether getting pregnant will enhance disease progression	1 (7.7%)	6.0 (0.0)
Not as mentally sharp	1 (7.7%)	4.0 (0.0)
Challenge of finding good doctors who understand disease	1 (7.7%)	9.0 (0.0)
Osteoporosis	1 (7.7%)	9.0 (0.0)

*LOPD* late-onset Pompe disease, *SD* standard deviation

*Denotes wording applied to third round of interviews only (Additional file 1)

^†^Impact disturbance rating was performed by three patients

**Fig. 2:**
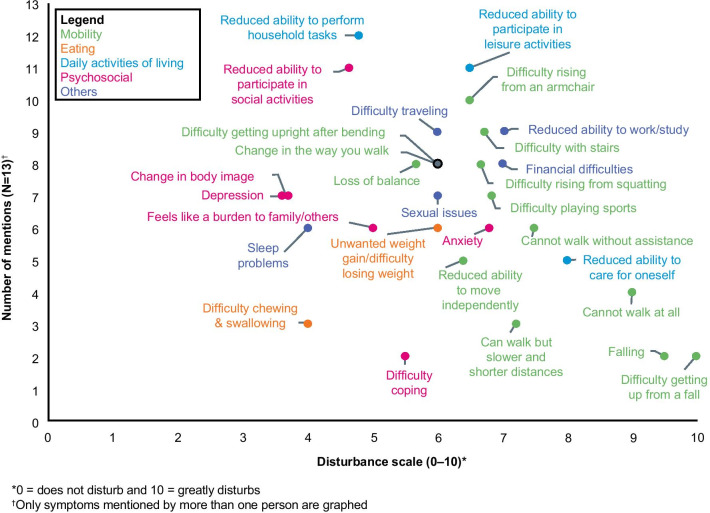
Summary of patient findings: impacts

In contrast to the most frequently occurring symptom impacts on patients, those which patients rated highest on the impact disturbance scale (mean [SD]) formed a different set: ‘difficulty getting up from a fall’ (10 [0.0]), ‘falling’ (9.5 [0.5]), ‘cannot walk at all’ (8.5 [1.7]), and ‘reduced ability to care for oneself’ (8.3 [1.2]). The patient-based evidence regarding symptom-related impacts likely to be relevant and important to patients with LOPD is shown in Fig. 2. Mobility impacts and how these affect daily activities appeared most frequently mentioned and were significantly affected by the disease, specifically, difficulty rising from an armchair and from a squatting position, difficulty climbing stairs, and a reduced ability to participate in leisure and work/study activities.

Saturation of LOPD symptom-related concepts was achieved after the second round of interviews. The majority of symptom-related concepts first appeared in Group 1 (19/23, 83%) followed by 17% (4/23) in Group 2. No new concepts were identified from Group 3 patient responses. Impact-related concepts showed a similar pattern. The majority (40/48, 83%) were identified in Group 1, followed by 17% (8/48) in Group 2, with none reported in Group 3.

Data obtained from patient interviews were triangulated with those gained from both the literature review and the expert interviews. Greater weight was given to patients’ reported concepts versus expert opinion because these had been directly experienced. An example of such is where experts considered sleep problems to be among the most significant impacts on patients’ lives, while patients considered it less disturbing than a variety of other impacts, such as mobility issues, limitations of work/study and some other activities of daily living, sexual issues, and financial difficulties. Concepts were also reworded to reflect patient rather than clinical language, for example, ‘Muscle weakness, especially in limb-girdle distribution (the leg and hip muscles)’ was reworded to read ‘Muscle weakness in lower body (legs and/or hips)’.

**Fig. 3:**
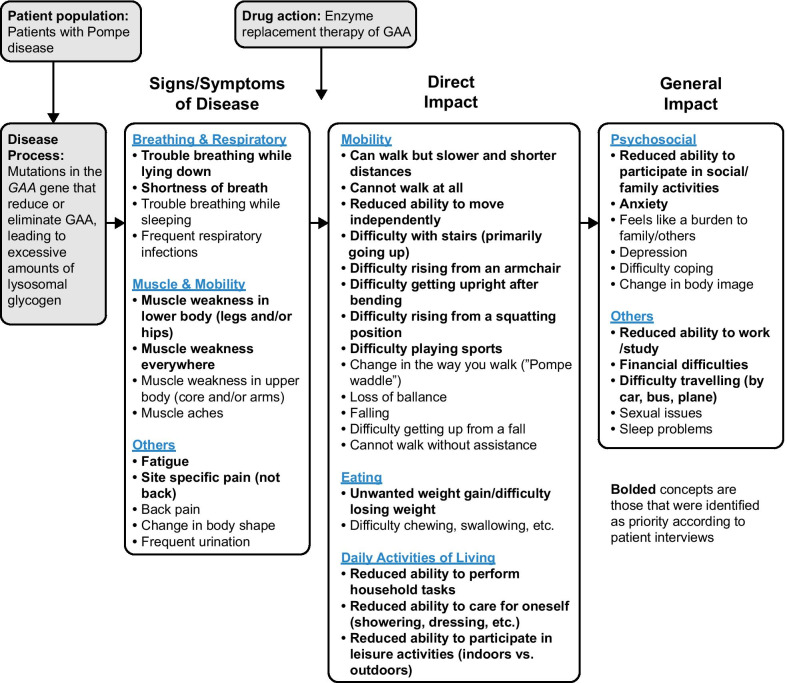
Finalized conceptual model

Post triangulation, the conceptual model was revised to encompass the symptoms of LOPD, as well as the direct and general symptom impacts of the disease on patients (Fig. 3). The model encompasses seven categories (Breathing and Respiratory, Muscle, Mobility, Eating, Daily Activities of Living, Psychosocial, and Other) and includes separate items under each category that reflect their relative importance in the lives of patients with LOPD. Muscle weakness, fatigue, breathing difficulties, and pain were confirmed as the most relevant symptoms whereas mobility problems, especially those associated with ADL, were confirmed as being most representative of how symptoms impacted on patients with LOPD.

Those specific symptoms and impacts considered high priority for measurement in any amended or future LOPD PRO instrument are shown in bold text in the conceptual model (Fig. 3) and include: trouble breathing while lying down; shortness of breath; muscle weakness; fatigue/tiredness/need to rest; pain; mobility issues such as difficulty walking, rising from sitting, or getting around; unwanted weight gain and difficulty losing weight; reduced ability to perform household and self-care tasks; reduced ability to participate in leisure, social, and family activities; anxiety/worry; reduced ability to work or study; difficulty traveling; and financial difficulties. Although not presented here, and with the exception of pain, an additional analysis of first signs of disease (pre-diagnosis) showed a similar pattern of symptom importance in patients (data on file).

## Discussion

The overall aim of this study was to identify the most important signs, symptoms, and functional limitations as experienced and directly reported by patients with LOPD. This perspective is critical to provide the information and evidence needed to support development of a novel PRO instrument for use in clinical trials. Currently, none of the existing PRO instruments for LOPD capture all of the symptom concepts, and use the patient-preferred language, identified in this study. Furthermore, the results reported here are based on interviews rather than patient surveys, therefore, future PRO instruments based on these results may provide a more accurate and up-to-date measure of the patient experience with LOPD. In addition, several key concepts that were frequently reported and/or disturbing to patients in this study, are not captured by currently available instruments, including back pain, muscle weakness (upper and lower body), falling, difficulty getting up from a fall, loss of balance, in addition to respiratory and breathing problems. Other PRO instruments previously used to assess alglucosidase alfa treatment of PD, such as the 36-Item Short Form Surveys (SF-36) [28, 29], are generic instruments which lack specificity for PD, whereas PROs that may be specific for certain symptoms, such as the Fatigue Severity Scale (FSS) [30], are not comprehensive to all of the domains impacted in PD. Furthermore, in considering clinical trial outcomes, none of the existing measures were fully aligned with FDA expectations for PRO instrument development anticipated to support product labelling [27, 31].

An essential step in the development of a novel conceptual model is talking to patients to understand their experiences. Through patient interviews, symptoms can be explored and their impacts on daily living can be better understood. Documentation of frequency, severity, and duration, along with determining specific words and phrases patients use to report symptoms and impacts, are fundamental to understanding patients’ experiences. Additionally, where patients provide written responses to non-verbal interviews, questionnaire forms should be uncomplicated with clear instruction. Instruments used to assess outcomes of importance and relevance to patients regarding treatment of symptoms and/or symptom impacts, must demonstrate validity, reliability, and the ability to detect change.

Despite the variability in LOPD symptoms, in the current study most patients experienced a similarly concentrated group of symptoms; however, the most frequent symptom impacts, and those reported as having a high level of disturbance on patients’ daily lives, were more widely distributed. The most frequent symptoms and those reported as having a high level of symptom disturbance on patients’ daily lives were fatigue, muscle weakness, pain, and breathing difficulties (especially while lying down). Of the total patients surveyed, five impact-related concepts were mentioned by more than 75% of patients, and 50–74% experienced 13 impact-related concepts; of these, almost half were in the Muscle and Mobility category. Patients reported a reduced ability to perform activities of daily living, (specifically household tasks and leisure activities), to work or study, or participate in social and family activities.

The combined approach of literature review, expert clinician interviews, and concept elicitation interviews with patients has not only generated a better understanding of the concepts relating to symptoms and impacts in LOPD as experienced by patients, but also an appreciation of how those symptoms and impacts affect patients in their daily lives. The conceptual model developed through this process identifies the most frequent and relevant symptoms and symptom impacts on patients for consideration in PRO measurement for LOPD.

The information obtained from this study may aid in the creation of a novel PRO instrument for patients with LOPD which can address the gaps in existing PRO tools. In particular, currently available PRO tools are limited by the type of symptoms or impacts they measure, such as the specific respiratory difficulties found in LOPD. One of the key findings of this study was that patients reported respiratory difficulties as one of the most frequent symptoms, with a corresponding significant rating on the symptom disturbance scale. These respiratory problems ranged from “shortness of breath” to difficulties in breathing “when lying down” and “while sleeping.” The inclusion of disease-specific PROs in clinical trials is necessary to capture the most comprehensive patient’s perspective of their health status, and to provide guidance for optimal management of the disease.

## Limitations

While the sample size of this study was too small to be truly representative of the entire LOPD population, efforts were made to align the patients interviewed with those who may qualify for participation in clinical trials. The fact that expert clinician and patient interviews aligned with the findings of the LOPD literature review indicates that the symptoms and impacts described in the conceptual model are representative of the range of experiences in patients living with LOPD. However, as all patients participating in this study were receiving ERT, the symptoms and impacts identified may not fully represent those in patients not currently receiving treatment.

## Conclusion

A conceptual model was created that provides patient interview-based evidence regarding the symptom-related concepts and impacts relevant and important to patients diagnosed with LOPD. Levels of associated symptom and impact disturbance to patients’ daily lives in this patient population were identified to better determine the magnitude of the daily effects on patients’ lives. The patient contribution of concepts critical to their experience with the disease is reflected in the results on symptoms, impacts, and their associated disturbance levels. This exploratory and conceptual investigation highlights the importance of ensuring all relevant symptoms and associated functional impacts experienced by patients and expressed in patient-preferred language are included in comprehensive PRO measurements in LOPD-specific clinical trials.

## Supplementary Information

**Additional file 1:** Change in text format from third round of interviews.

**Additional file 2:** Patient Quotations.

## Data Availability

Qualified researchers may request access to patient level data and related study documents. Patient level data will be anonymized and study documents will be redacted to protect the privacy of trial participants. Further details on Sanofi’s data sharing criteria, eligible studies, and process for requesting access can be found at: https://www.clinicalstudydatarequest.com.
