# Supplementary material for: Qualitative interviews to improve patient-reported outcome measures in late-onset Pompe disease: the patient perspective
Source: Orphanet J Rare Dis. 2021 Oct 12;16:428. doi: 10.1186/s13023-021-02067-x (PMC8513325; doi:10.1186/s13023-021-02067-x)
Supplement: Supplementary file 1 — Additional file 1: Change in text format from third round of interviews. [file 13023_2021_2067_MOESM1_ESM.docx]

**Additional file 1:** Change in text format from third round of interviews

| **Wave 1 and Wave 2 item wording** | **Wave 3 item wording alterations** |
| --- | --- |
| Muscle weakness in upper body (core and/or arms) | ***Muscle weakness in upper body (neck, back, trunk, and/or abdomen)*** |
|  | ***Muscle weakness in arms*** |
|  | ***Muscle weakness in the grip of your hand*** |
| Trouble breathing while lying down | ***Breathing difficulties while lying down*** |
| Shortness of breath | ***Breathing difficulties*** |
| Fatigue | ***Tiredness/fatigue/need to rest*** |
| Site specific pain (not back) | ***Pain*** |
| Headache | ***Morning headache*** |
| Difficulty rising from an armchair | ***Difficulty rising from a sitting position*** |
| Difficulty with stairs (primarily going up) | ***Difficulty climbing stairs*** |
| Cannot walk without assistance | ***Can walk with assistance (from another person or a device, like a cane or walker)*** |
| Unwanted weight gain / difficult losing weight | ***Unwanted weight gain*** |
|  | ***Difficulty losing weight*** |
| Reduced ability to participate in social activities | ***Reduced ability to participate in social/family activities*** |
