# Supplementary material for: Qualitative interviews to improve patient-reported outcome measures in late-onset Pompe disease: the patient perspective
Source: Orphanet J Rare Dis. 2021 Oct 12;16:428. doi: 10.1186/s13023-021-02067-x (PMC8513325; doi:10.1186/s13023-021-02067-x)
Supplement: Supplementary file 2 — Additional file 2: Patient Quotations. [file 13023_2021_2067_MOESM2_ESM.docx]

**Additional file 2**: Patient quotations

The examples below illustrate salient concepts highlighted in the final model.

1. Symptoms

**Fatigue concept:** “Probably the worst one [symptom] would be fatigue. That’s always there...Feeling tired all the time. I’ve described it to people when I wake up in the morning, I can have nine or ten hours of sleep and I still am almost as tired as when I went to sleep. So I never feel well rested."

**Muscle weakness concept:** “I say it’s a muscle weakening disease…Muscle weakness is always there. Like it’s impossible to bend over and pick up something from the floor and stand up again. Even if it’s a tissue or something very light. Doesn’t need to be something heavy. Or to get down on the floor and stand up. That is the muscle weakness issues. Even walking is muscle weakness in my eyes. I don’t always feel pain when I walk, but I can definitely feel a weakness in my walking.”

**Trouble breathing when lying down concept:** “When I go to sleep or when I lay down it feels like [something is] on my chest you know and it’s just, it’s hard to breathe.”

**Shortness of breath concept:** “I was actually just having breathing problems, which had been falsely diagnosed as asthma. You just feel like you’re not getting enough air, I guess. I feel like I can’t breathe as well as I would normally, and then sometimes it will lead to wheezing.”

**Pain concept:** “The stabbing and shooting pains are usually in my legs and feet, sometimes in my arms, and there’s a lot of achy pain in my back. Mostly my lower back, but sometimes it’s all over. And when I fall, my tailbone usually hurts for a good three, four, five days afterwards. Muscle aches are all over. It’s definitely worse in the evening, after I’ve done whatever I’ve done throughout the day. If I’m overly active, if I push myself too hard, I usually suffer for that night and the next day.”

1. Impacts
   1. Mobility concept

**Walking limitations:** “I stopped walking at all…About 10 or 11 years ago. I used a walker and a wheelchair but then I fell down so many times finally I broke my hip and then that was my, you know, cue to stop walking and use the power chair.”

**Assistance concept:** “I can walk probably 40 or 50 feet. But then you need to stop, gain your breath and go on. I use a walker around the house here. I use scooters when I go outside to doctor’s appointments. I can move a little by myself, but I have to have a walker or a cane. I’m slow at it …because I don't take a chance of falling. I just don’t take that chance anymore."

**Independent movement concept:** “Just like I say, loss of some independence when it comes to just doing daily things even. I’ve had to learn to be very patient and ask for help and wait for others to help me whereas I’ve always been very independent and be able to do everything myself. Now, you can’t and you have to learn to be a lot more patient and that took probably two to three years to learn that I just have to be patient and eventually it will get done."

**Difficulty with stairs:** “We moved last year from a two-story house to a one-story house because climbing stairs is... I can only do like one flight of stairs or a couple flights of stairs a day. It’s just a real effort to go up and down the stairs. And then it’s also difficult to walk uphill. So, yeah, climbing stairs is difficult."

**Rising from armchair:** “Getting up out of a chair is very difficult. I just can’t stand up. I have to use my arms which means I put them on the table or put them on another chair and use my cane.”

**Getting upright after bending:** “I can’t bend very far or I’ll be down on the ground… If I have to pick anything up, it’s a struggle. I have to use a grabber or make sure I’m close to something that I can put my arms on to bend over and then get back up.”

**Playing sports:** “I played volleyball a lot and I really don’t play at all anymore. Again, I can’t keep up. I’m not physically able to do that."

- 1. Reduced participation in social, leisure, family, work/study activities:

**Leisure concept:** “I used to be very active so I used to sail and hike and backpack and, of course, diving...I can’t do any of that anymore.”

**Social/family concept:** “I certainly can’t do what I used to do. I have a 12-year-old son, who I can’t do a lot of things with him... Activities, when I am playing ball with him or riding a bike with him, things like that. Those things I can’t do. I can’t be too active. […] And so it’s my wife, too. I can’t do any chores pretty much around the house.”

**Work/study concept:** “I am unable to work now at the job I was currently doing before diagnosis so I’m on disability. So that affects me financially because I don’t make near as much as I did before.”

#### Limits on activities of daily living:

**Household tasks concept:** “I can’t wash the kitchen floor or clean the shower or bathtub, so I just give the job to somebody else, which is fine. Like certain things where I can’t do anything, I always say below knee level. I can’t get down and do anything that is cleaning down low or actually up high. I couldn’t clean something up high on the ceiling or windows. That goes to another person.”

**Self-care concept:** “I need help dressing, I need help washing, getting to the bathroom. I’m okay once I am there, but getting there…”

**Difficulty traveling concept:** “My friends can take their car. I just can’t get into the car with them and go with them. Everything has to be so planned precisely. Even taking buses I have to take two buses. Everything has to be scheduled, bus stops, this way and this way. That is kind of one of the things that impacts my life. Being in a power chair is wonderful except that you can’t just get into a regular car.”

**Financial concept:** “It adds extra financial burden to a lot of things. Right now I need a new vehicle to drive, but for me to get a new vehicle requires a lot of customization just to make it so that I can steer and stop, which costs an extra 40 grand, so financially it makes it harder. Right now I’m on disability.”

**Anxiety concept:** “I avoid a lot of situations, sometimes, because I don’t want to be the center of attention. And people are so nice, they’re always trying to help you even when I don’t need help and that creates anxiety and embarrassment to some degree. […] The thought of other people having to take care of me doesn’t terrify, me but it really makes me anxious. I really… I don’t want to see that but… it’s starting to sink in now that, you know what, I might not be walking next year at this time and it does make me anxious."

**Weight gain/difficulty losing weight concept:** “A Pompe pouch, a lot of people call it that. Where we get a little bit of a bloated belly. Part of that, I thought was my age, too. Gaining more weight around my midsection… This started happening more around the time I was being diagnosed and soon after treatment, I started noticing more weight gain. Hard to say if it is age-related or not.”
